# Controlled Photocatalytic Synthesis of Core–Shell SiC/Polyaniline Hybrid Nanostructures

**DOI:** 10.3390/ma9030201

**Published:** 2016-03-16

**Authors:** Attila Kormányos, Balázs Endrődi, Róbert Ondok, András Sápi, Csaba Janáky

**Affiliations:** 1 Department of Physical Chemistry and Materials Science, University of Szeged, 6720 Szeged, Hungary; attila.kormanyos@chem.u-szeged.hu (A.K.); endrodib@chem.u-szeged.hu (B.E.); ondokrobert@freemail.hu (R.O.); 2 MTA-SZTE ”Lendület” Photoelectrochemistry Research Group, Rerrich Square 1, 6720 Szeged, Hungary; 3 Department of Applied and Environmental Chemistry, University of Szeged, 6720 Szeged, Hungary; sapia@chem.u-szeged.hu

**Keywords:** photocatalysis, electrochemistry, hybrid materials, semiconductor, heterojunction, conjugated polymer, optoelectronics

## Abstract

Hybrid materials of electrically conducting polymers and inorganic semiconductors form an exciting class of functional materials. To fully exploit the potential synergies of the hybrid formation, however, sophisticated synthetic methods are required that allow for the fine-tuning of the nanoscale structure of the organic/inorganic interface. Here we present the photocatalytic deposition of a conducting polymer (polyaniline) on the surface of silicon carbide (SiC) nanoparticles. The polymerization is facilitated on the SiC surface, via the oxidation of the monomer molecules by ultraviolet-visible (UV-vis) light irradiation through the photogenerated holes. The synthesized core–shell nanostructures were characterized by UV-vis, Raman, and Fourier Transformed Infrared (FT-IR) Spectroscopy, thermogravimetric analysis, transmission and scanning electron microscopy, and electrochemical methods. It was found that the composition of the hybrids can be varied by simply changing the irradiation time. In addition, we proved the crucial importance of the irradiation wavelength in forming conductive polyaniline, instead of its overoxidized, insulating counterpart. Overall, we conclude that photocatalytic deposition is a promising and versatile approach for the synthesis of conducting polymers with controlled properties on semiconductor surfaces. The presented findings may trigger further studies using photocatalysis as a synthetic strategy to obtain nanoscale hybrid architectures of different semiconductors.

## 1. Introduction

Hybrid materials, based on conducting polymers (CP) and inorganic materials, attracted a lot of interest in the past two decades [1,2]. Nanocomposite materials, exploiting the synergistic combination of the CP and the incorporated component (for example, metal nanoparticles, carbon nanotubes, and inorganic semiconductors (SC)) have been synthesized [3,4,5,6]. Hybrid assemblies of CPs and inorganic SCs provide the possibility of constructing high quality CP/SC interfaces, enabling a wide range of promising applications, such as photocatalysis, solar cells, sensors, and supercapacitors [7,8]. A wide array of synthetic approaches have been deployed, from simple mechanical mixing and *in situ* chemical polymerization, to electrochemical methods [9]. As shown in our recent review article, however, sophisticated synthetic protocols are indeed necessary to obtain sufficient control over the composition and morphology [9]. 

Silicon carbide (SiC) is a wide bandgap SC (*E_BG_* = 2.4–3.1eV, depending on which polymorph we are looking at) [10] with excellent physical and chemical properties, such as good mechanical and chemical stability, biocompatibility, photoluminescent behavior, high thermal conductivity and, last but not least, an affordable price [11,12,13,14,15]. Thus, SiC has been employed in a wide range of applications, for example, as substrate for high-temperature and high-power electronics [16], spintronics [17], optoelectronic devices [17] and quantum information processing [18]. As another prominent application area, SiC nanoparticles have been used in photocatalytic environmental remediation, for example, to degrade acetaldehyde [19] as a model compound. In addition, the conduction band edge position of SiC allows this material to photocatalytically reduce water or CO_2_ (in the presence of a sacrificial electron donor), which was first demonstrated in 1979 [20].

SiC-based hybrid materials offer an attractive avenue to simultaneously harness the eminent properties of SiC as well as those of the other component. In this vein, SiC has been already combined with different CPs, such as polypyrrole (PPy) [21,22], polyaniline (PANI) [5,23], and poly(3-thiophene-acetic-acid) [24]. PANI is particularly attractive, because of its versatile redox behavior, excellent chemical and electrochemical stability, large capacitance, and electrochromic properties [25,26,27]. The commonality in these reports is that every synthesis was carried out by *in situ* oxidative *chemical* polymerization, differing only in the oxidizing agent employed (e.g., FeCl_3_). In such procedures the oxidant reacts with the monomer to form a radical cation, which initiates the polymerization by reacting with another monomer or radical cation. This method, however, gives only very limited control over the structure and morphology of the synthesized composite material. On the other hand, to efficiently harness the possible synergies of the components, the precise control over composition, morphology and thus the interfacial properties is inevitable. Consequently, there is a massive demand for new synthesis strategies, which allow hybrid materials with properties tailored towards specific applications to be obtained.

Photocatalysis, occurring at the SC/electrolyte interface is a broadly studied phenomenon [28], mostly in the field of solar energy conversion and environmental remediation. On the other hand, much less attention is devoted to its utility as a synthesis tool. Biomass valorization using photocatalysis is a promising example [29], but other areas still have to be explored. The limited number of studies on the photocatalytic synthesis of hybrid materials of inorganic SCs and organic CPs is indeed surprising, considering the photoactivity of both components. The most important beneficial feature of this approach is that the CP is formed *in situ*, on the surface of the inorganic SC nanostructure, thus bringing the two SCs into intimate contact [30]. 

The pioneering studies were performed on colloidal, or nanoparticulate, titanium dioxide (TiO_2_) slurries [31,32], using pyrrole as the monomer. In these cases, the nanoparticles were dispersed in the monomer- and supporting electrolyte-containing solution. The dispersion was exposed to ultraviolet (UV) irradiation under continuous stirring and saturation with O_2_ (which acts as electron scavenger). As the result of polymerization, a thin PPy film was deposited on the surface of TiO_2_. Follow-up studies demonstrated the suitability of this method using other SCs, such as cadmium sulfide (CdS) [33], cadmium telluride (CdTe) [33], cadmium sulfide@zinc sulfide CdSe@ZnS [34], and polymers (e.g., poly(3,4-ethylenedioxytiophene)-PEDOT) [35], and also yielded further mechanistic details [36]. Finally, we note that two sophisticated varients of this approach were also demonstrated: (i) photoelectrochemical polymerization (where external electrical bias is employed instead of a chemical electron scavenger) [9,37]; and (ii) the photocatalytic deposition of CdS on the surface of poly(3-hexylthiophene) (in this case, the photoactivity of the CP is exploited in a similar manner) [38].

In this study we add another composite material, namely SiC/PANI, to the library of photocatalytically prepared hybrid assemblies. A carefully designed synthetic protocol is presented, which results in core–shell nanostructures, with a high quality organic/inorganic junction. Finally, it is demonstrated that it is possible to tune the composition of the composite material by simply changing the irradiation time.

## 2. Materials and Methods 

### 2.1. Chemicals

For the nanocomposite synthesis, SiC (U.S. Research Nanomaterials Inc, Houston, TX, USA average diameter ≈ 50 nm), aniline (Sigma-Aldrich), and sulfuric acid (95%–98%, Sigma Aldrich, Sigma Aldrich, Budapest, Hungary) were used, along with O_2_ gas (99.995%, Messer, Budapest, Hungary). For the preparation of the dispersions used for spray coating, Nafion solution (10%, Fuelcellstore.com) was employed. All chemical reagents were of analytical grade and were used without further purification, except for the aniline monomer, which was freshly distilled before use. Deionized water (MilliPore, 18 MΩ) was used to prepare all solutions.

### 2.2. Nanocomposite Synthesis

A solution (*V_total_* = 5 mL), containing 0.2 M aniline, 10 mg SiC nanoparticles, and 0.5 M H_2_SO_4_ was prepared for each synthesis. In the two sets of experiments (with or without hard UV irradiation), the slurries were put in a quartz or Pyrex^®^ photoreactor, (custom designed and manufactured in Hungary) depending on the type of illumination (Pyrex^®^ absorbs all the components of the irradiating light below 300 nm, while quartz transmits the whole spectrum). The dispersion was constantly stirred and saturated with O_2_ during the synthesis, and illuminated by a 300 W Hamamatsu L8251 Hg-Xe arc lamp (Hamamatsu Photonics, Hamamatsu, Japan). The time of irradiation was varied (5, 10, 30, and 60 min). After the core–shell nanocomposite synthesis, the samples were centrifuged with a VWR 1814 centrifuge, then washed and centrifuged consecutively two more times. Finally, the samples were dried under vacuum at 80 °C for 4 h.

For the electrochemical characterizations, the samples were spray-coated on an Au electrode surface. The dispersion contained 2 mg/mL nanocomposite (or pristine SiC) and 25% Nafion^®^. The mass of the material on the electrode surface was approximately 2 mg in each case.

### 2.3. Characterization

Thermoanalytical measurements (thermogravimetry-TG and differential thermogravimetry- dTG) were performed on a TA Q800 thermogravimetric analysis (TGA) instrument. In a typical experiment, 3–5 mg of a sample was heated in synthetic air (20.5% O_2_ in N_2_) from room temperature to 800 °C with a heating rate of 10 °C/min. Raman spectroscopy was performed on a DXR Raman microscope (Thermo Scientific, Waltham, MA, USA) using a *λ* = 532 nm green laser with 10 mW laser power. Attenuated total reflectance Fourier transformed infrared spectroscopy (ATR-FT-IR) studies were performed using a Bio-Rad Digilab Division FTS-65A/896 Fourier transform infrared spectrometer (Bio-Rad Laboratories, California, CA, USA), equipped with a Harrick’s Meridian^®^ SplitPea single-reflection diamond attenuated total reflectance (ATR) accessory. All presented spectra are an average of 256 interferograms with 4 cm^−1^ optical resolution. Transmission electron microscopic (TEM) images were taken at various magnifications on a FEI Tecnai G^2^ 20 X-Twin instrument (FEI, Hillsboro, OR, USA), operating at an acceleration voltage of 200 kV. For the scanning electron microscopy (SEM), a Hitachi S-4700 FE-SEM instrument (Hitachi, Tokoy, Japan) was used. Cyclic voltammetric experiments were carried out on a PGSTAT 10 (Autolab) instrument (Metrohm Autolab, Kanaalweg, Netherlands). A classical, sealed three-electrode cell was used, purged with Ar gas during the measurements. An Au foil, spray-coated with the hybrid materials, was used as a working electrode (A = 1 cm^2^). A Pt foil and a Ag/AgCl/3M NaCl were used as counter and as reference electrodes, respectively. The measurements were carried out in 0.5 M H_2_SO_4_ at a 25 mV/s sweep rate.

## 3. Results and Discussion

### 3.1. Synthesis of the Core–Shell Nanocompostites

**Figure 1:**
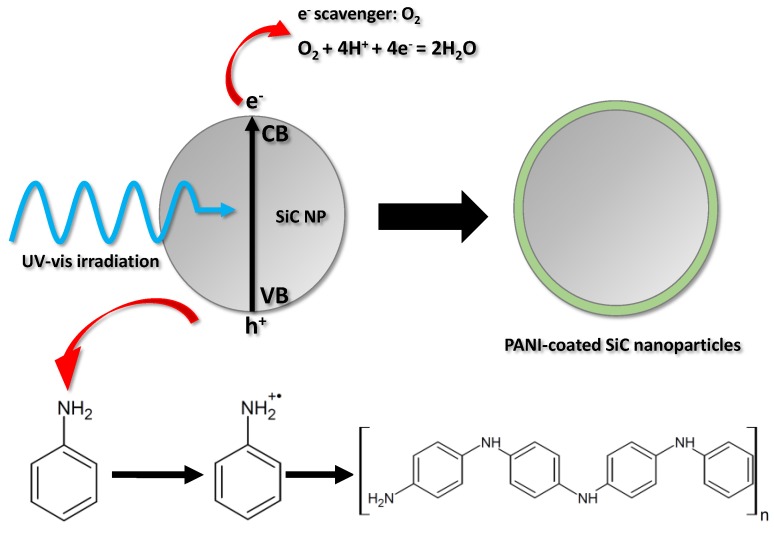
Schematic illustration of the nanocomposite synthesis.

The schematic illustration of the nanocomposite synthesis is given in Figure 1. The SiC nanoparticles were dispersed in the solution of the aniline monomer and the sulfuric acid supporting electrolyte, and stirred constantly, ensuring that the incident photon flux evenly reached the surface of the dispersed SiC nanoparticles. As a result of ultraviolet-visible (UV-vis) light irraditation, electron-hole pairs are formed in the SiC nanoparticles. After charge separation, some of the photogenerated charge carriers reach the particle surface before they could recombine. These charge carriers can initiate various reactions at the SiC/electrolyte interface. In our case, the holes oxidize the aniline monomers, generating radical cations. These first form oligomers in the solution, close to the surface of the SC nanoparticles and, thereafter, a continuous ultrathin polymer coating is deposited on the surface. Contrastingly, the photogenerated electrons react with dissolved O_2_, which is present in the solution as an e^−^ scavenger. The polymerization can be terminated in two ways: (i) by turning off the illumination; (ii) or by coating the surface of the SiC nanoparticles evenly (without any gaps) with the gradually thickening PANI film, which causes optical shielding by absorbing the irradiating light.

**Figure 2:**
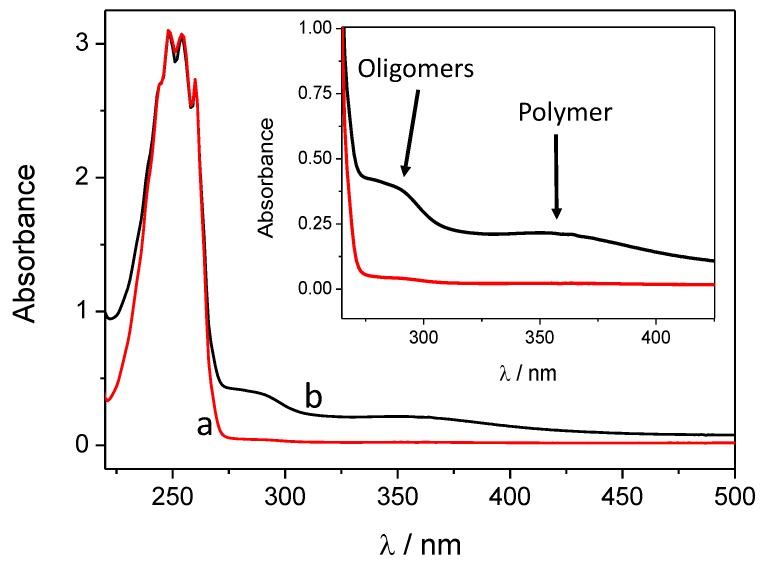
Ultraviolet-visible (UV-vis) spectra of the filtered and centrifuged dispersion after 60 min of illumination: (**a**) hard ultraviolet (UV)-filtered, (**b**) hard UV-illuminated.

The first spectacular difference between the two synthetic methods (*i.e.*, with and without hard UV light irradiation) is manifested in the color of the solution after centrifuging the SiC/PANI nanoparticles. The first, irradiated with the full white light irradiation, turned dark red, while the second one remained almost transparent. After centrifuging and filtering, the UV-vis spectra of the two residual solutions were measured (Figure 2). Beyond the obvious low-wavelength band of the monomer, two wide bands appeared in the case of the hard UV-illuminated sample: one corresponds to the formed oligomers with low segment number, the other one to the polymer fragments in the solution [39]. Contrastingly, on the spectrum recorded for the UV-filtered sample, no band appears which could correspond to the polymer/oligomers. There are at least three possible factors contributing to these observations: (i) because of the hard UV illumination, more polymer forms and the excess stays in the solution causing the dark reddish color; (ii) when the hard UV illumination occurs, the aniline is photopolymerized in the solution; (iii) finally, it is also possible that the high energy UV illumination degrades PANI, which is already formed on the SiC surface of the nanoparticles, which may therefore leach into the solution. 

**Figure 3:**
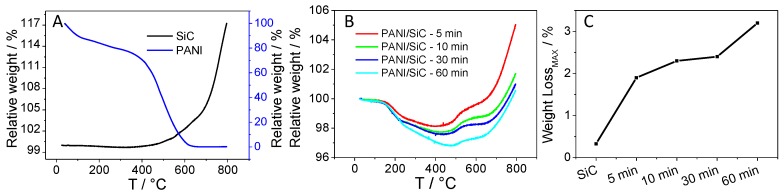
Weight loss curves registered during the thermogravimetric analysis (TGA) for (**A**) silicon carbide (SiC) and polyaniline (PANI); (**B**) and the various SiC/PANI hybrids; (**C**) a comparison of the PANI content of the various SiC/PANI hybrids as derived from TGA.

Thermogravimetric analysis (TGA) was employed to determine the amount of the formed PANI after certain irradiation time periods, and thus to assess the composition of the various nanocomposite samples. As a first step, the pure SiC was studied, where an 18% mass increase was observed between ~330 °C–800 °C, attributed to the oxidation of SiC nanoparticles (the black line in Figure 3A). As for the pure PANI sample, two major decomposition steps occurred in the range of 25 °C–660 °C. In the first step (25 °C–275 °C), the weight loss of 22% can be ascribed to the water and SO_x_ desorption (from the HSO_4_^−^ dopant ions) from the sample. In the second step (275 °C–660 °C), the complete oxidation of the polymer backbone was observed (the blue trace in Figure 3A) [40].

For all SiC/PANI composites, a two-step weight loss occurred in the range of 25 °C–~485 °C attributed to the thermal degradation of PANI. This is followed by a two-step weight increment due to the oxidation of SiC in the range of 400 °C–800 °C. The TG curves are the superposition of those registered for the pure materials in Figure 3A. In the first step (25 °C–250 °C), the water and dopant content desorbed, followed by the degradation of the PANI polymer chain in the range of ~250–~500 °C. As the irradiation time was increased from 5 min to 60 min, the maximum weight loss (at elevated temperatures) increased from 1.9% to 3.2%, showing the gradually growing portion of PANI in the composites. In addition, at elevated temperatures (500 °C–800 °C), the oxidation of the SiC occurred in a two-step process, as opposed to pure SiC, where only one-step oxidation was witnessed. We suspect that some carbon-based residual of PANI may influence the oxidation and sintering of the SiC nanoparticles. 

### 3.2. Structural Characterization

#### 3.2.1. Raman Spectroscopy

**Figure 4:**
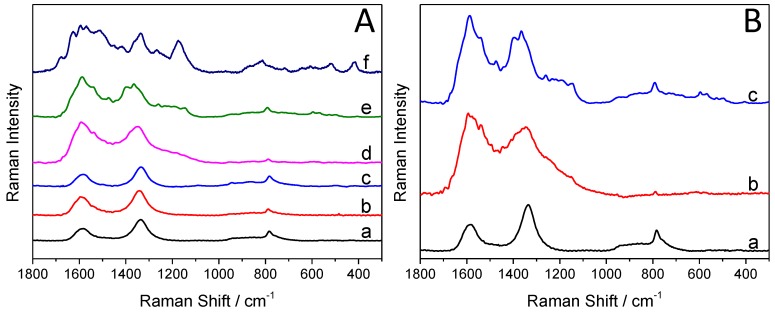
(**A**) Raman spectra of the pure silicon carbide (SiC) and PANI, and the SiC/PANI nanocomposites (with the UV component filtered below 300 nm): (**a**) SiC, (**b**) SiC/PANI, 5 min of illumination, (**c**) SiC/PANI, 10 min of illumination, (**d**) SiC/PANI, 30 min of illumination, (**e**) SiC/PANI, 60 min of illumination, (**f**) PANI; (**B**) Raman spectra of the synthesized nanocomposite samples: (**a**) SiC, (**b**) SiC/PANI 60 min of illumination, UV-illuminated, (**c**) SiC/PANI 60 min of illumination, UV-filtered.

Figure 4A compares and contrasts the Raman spectra of the synthesized hybrid core–shell SiC/PANI nanoparticles (the irradiation was cut below 300 nm), the bare SiC, and the pure PANI. There are three strong bands on the Raman spectra of the SiC nanoparticles (Figure 4A, curve ”a”). The one, centered at 790 cm^−1^, is attributed to the lattice vibration of the material, specifically to the zone-center transverse optical (TO) phonons of 3C-SiC [41]. There are two additional characteristic bands, centered at 1334 cm^−1^, and at 1595 cm^−1^, corresponding to the vibrational properties of isolated carbon arrangements [41,42], indicating the existence of an ultrathin carbon film on the surface of the SiC nanoparticles.

The characteristic Raman bands of PANI are summarized in Table 1. The bands, appearing on the spectra of the nanocomposite samples (Figure 4A), are marked with grey. It is visible in the series of spectra (b–e) that with increasing illumination time, the quantity of the deposited polymer increases as well. Note that the intensity ratio of the bands, centered at 1334 cm^−1^, and 1595 cm^−1^ completely flipped over (because these bands are overlapping with those of PANI). In addition, most of the characteristic bands of PANI appeared on the spectra of the nanocomposite, proving the formation of the SiC/PANI hybrid. When the hard UV component of the illumination was not removed, some of the characteristic bands completely vanished, some others can still be identified (but with much smaller intensities (see the comparison in Figure 4B)).

#### 3.2.2. Infrared Spectroscopic Studies

**Figure 5:**
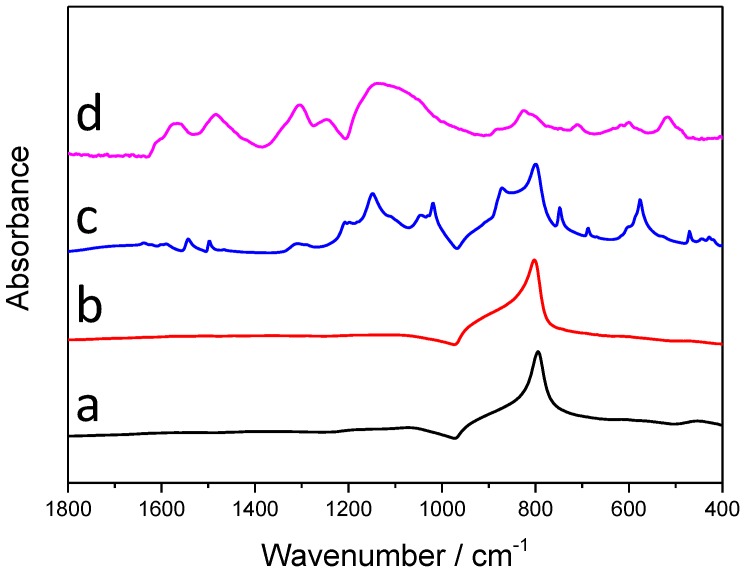
Infrared (IR) spectra of the synthesized nanocomposite samples and their components: (**a**) SiC; (**b**) SiC/PANI, 60 min UV-illuminated; (**c**) SiC/PANI, 60 min UV-filtered; (**d**) bare PANI.

The infrared (IR) spectra of the synthesized samples with the longest irradiation time (60 min) are presented in Figure 5. On the spectra of SiC (the black curve), only one sharp, characteristic band is visible, which corresponds to the Si-C stretching vibration, and there is an additional, rather weak band, centered around 1090 cm^−1^, which can be assigned to both the stretching vibration of Si-O-C and Si-O-Si bonds [24]. Table 2 contains the characteristic IR vibrations of PANI, with the bands appearing on the spectra of the SiC/PANI nanocomposites, marked with grey. Note that on the spectrum of the UV-illuminated composite there are no characteristic PANI peaks, which may indicate that the deposited polymer layer is overoxidized. Contrastingly, almost all the characteristic bands of the PANI appear on the spectra of the UV-filtered sample, which is another direct evidence that this milder synthesis procedure results in SiC/PANI nanocomposites containing high-quality PANI. 

### 3.3. Morphological Characterization

#### 3.3.1. Transmission Electron Microscopic Studies

**Figure 6:**
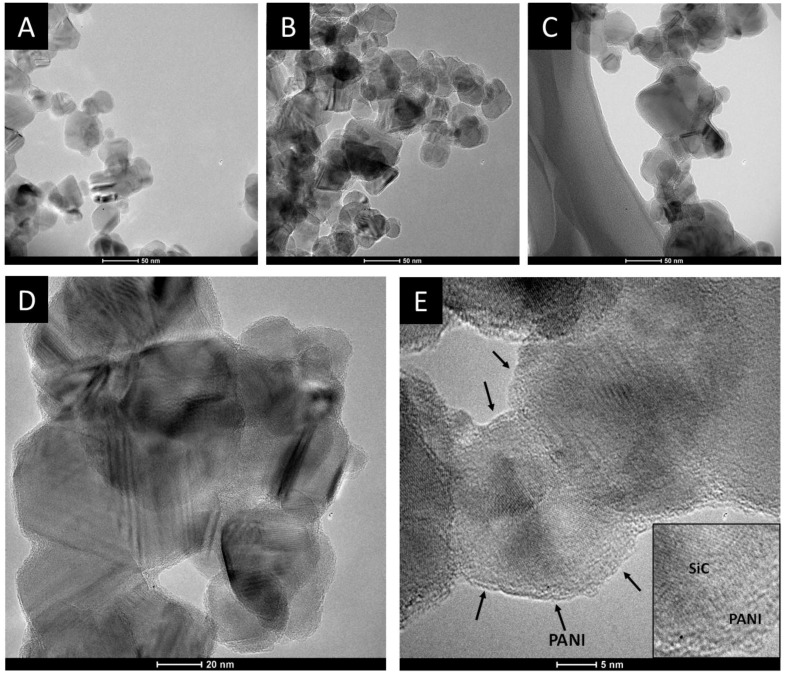
Transmission electron microscopy (TEM) images, captured for (**A**) SiC; (**B**) SiC/PANI, after 5 min of illumination; (**C**) SiC/PANI, after 60 min of illumination at 130,000x magnification; HR-TEM images taken for the SiC/PANI nanocomposite synthesized with 60 min of irradiation. The magnifications were (**D**) 255,000x; and (**E**) 530,000x, respectively.

The morphological characteristics of the SiC/PANI samples were first investigated by TEM. Figure 6 shows the TEM images recorded for the pristine SiC and for the SiC/PANI nanocomposites, synthesized with the shortest (5 min) and with the longest (60 min) irradiation time. The first conspicuous difference between the SiC and the nanocomposite samples, which can be observed on the images captured at a smaller magnification, is that the degree of aggregation is higher in the PANI-containing materials, despite the thorough mixing during synthesis. The images, taken at higher magnification, allow us to visualize both the lattice fringes of the SiC nanoparticles and the deposited PANI as a few nm-thick amorphous film on the surface of the nanoparticles (NP). Note that in the case of the pristine SiC, the lattice fringes are visible till the edges of the nanoparticles, but in the case of the nanocomposites an outer shell can be spotted, corresponding to the formed PANI film. 

**Figure 7:**
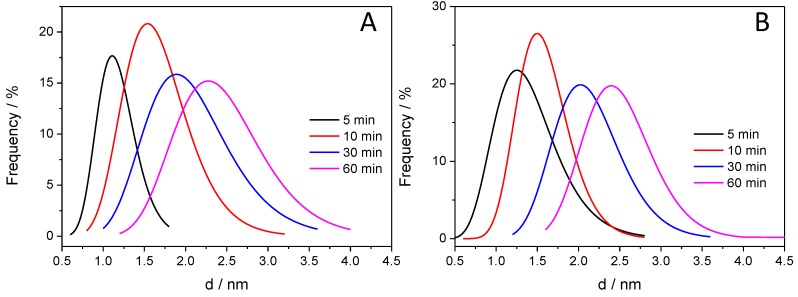
Distribution of the PANI layer thickness for the various SiC/PANI hybrids: (**A**) UV-irradiated samples; (**B**) UV-filtered samples.

After taking the images with the same magnifications for all the other samples, the thickness of the deposited polymer layer was determined. Figure 7 shows the distribution of the PANI shell thicknesses, thus allowing for quantitative comparison. It is visible that with increasing illumination time, the average film thickness is increasing from 1.0 to 2.5 nm, indicating that the thickness of the deposited PANI can be regulated by simply varying the illumination time. In addition, the forming PANI also glues together the individual SiC nanoparticles, as seen in the High Resolution Transmission Electron Microscopy (HR)-TEM images (Figure 6D,E). Importantly, there is no major difference between the thickness of the PANI layers synthesized with different light irradiations (see above).

#### 3.3.2. Scanning Electron Microscopic Studies

**Figure 8:**
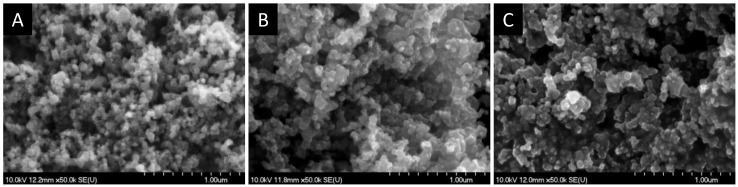
Scanning electron microscopy (SEM) images, taken for (**A**) SiC; (**B**) SiC/PANI UV-irradiated sample (60 min); (**C**) SiC/PANI UV-filtered sample (60 min).

SEM images were taken to characterize the overall morphology of the samples. Figure 8 shows SEM images for SiC and for the two hybrid SiC/PANI samples, synthesized with the longest irradiation time (60 min), differing only in the type of irradiation. As shown in Figure 8, the pristine SiC sample has a granular morphology, where the individual nanoparticles can be spotted. Contrastingly, in the other two images (Figure 8B,C), a more coherent structure is seen, where the polymer covers the SiC nanoparticles. In addition, it seems that PANI deposits not just on the surface, but also among the nanoparticles, thus gluing them together.

### 3.4. Cyclic Voltammetric Analysis

**Figure 9:**
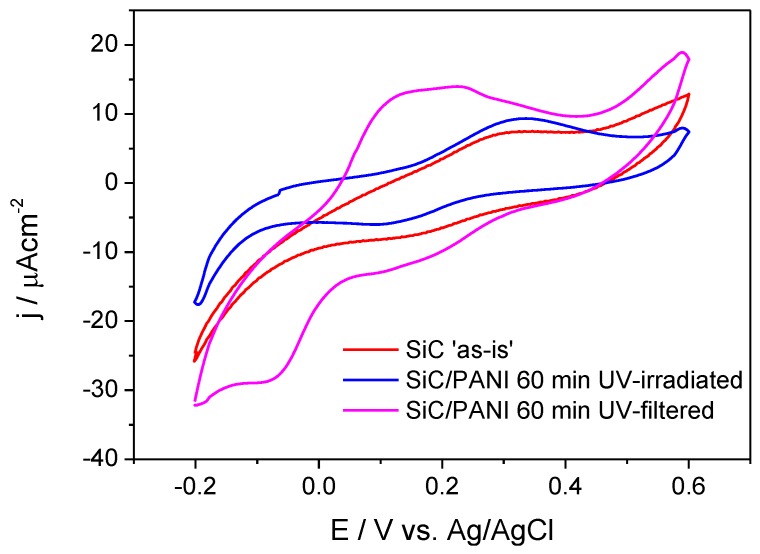
Cyclic voltammograms of the SiC nanoparticles and the synthesized nanocomposites recorded in 0.5 M H_2_SO_4_ at a sweep rate of 25 mV/s.

The electrochemical behavior of the samples was probed by cyclic voltammetry (Figure 9). It is visible that SiC has only limited electroactivity in this potential window (the red curve), which is comparable with the current densities measured for the SiC/PANI sample prepared under full UV irradiation. The shape of this latter voltammogram (the blue curve), however, is more pronounced: it has one anodic and one cathodic peak. If the synthesis procedure was milder (UV-filtered), the shape of the voltammogram clearly exhibits the features of the electrochemical transformation of PANI [26,27]. The most important conclusions to be drawn from these observations is that the SiC/PANI nanocomposite, prepared with filtering all the hard-UV component of the irradiation, was much more electroactive compared to its counterpart prepared using the full output of the lamp. This observation is in perfect agreement with the vibrational spectroscopic data presented above, namely that the hard UV component degrades the conjugated structure of PANI.

## 4. Conclusions 

In this study we demonstrated how photoexcitation of SiC nanoparticles can be used to deposit a conductive PANI coating on the particles’ surface. The presented photocatalytic polymerization is a facile and versatile approach to obtain hybrid organic/inorganic semiconductor assemblies. The only requirement is that the valence band edge of the semiconductor (e.g., TiO_2_ or CdS) is positive enough, so that the photogenerated holes can oxidize the monomer molecules to initiate (and sustain) the polymerization. As it was shown in the example of SiC/PANI, the thickness of the polymer shell can be varied between 1.0 nm and 2.5 nm (as confirmed via TEM analysis), simply by changing the irradiation time. This increasing PANI content was also corroborated by the TGA data, which additionally confirmed that there is no bulk PANI in the hybrid samples. As gleaned from vibrational spectroscopic and electrochemical studies, it was key to filter out the irradiation below 300 nm during synthesis. While polymer formation was evidenced even when the full UV-vis output of the lamp was utilized, the formed polymer was of low quality, both in terms of structure and electroactivity. This effect is mostly related to the formation of insulating polymer upon direct photopolymerization (*i.e.*, not by the photogenerated holes in SiC), as well as to the degradation caused by the high energy irradiation. In contrast, such problems did not occur when the hard UV component was filtered out during the synthesis. Overall, this study adds to the library of hybrid organic/inorganic materials obtained via photocatalytic synthesis. We hope that the presented results will encourage other researchers to exploit this attractive method to synthesize such hybrid nanoarchitectures with controlled composition and morphology. 

## Figures and Tables

**Table 1:** Characteristic Raman bands of PANI. The bands appearing on the spectra of the SiC/PANI nanocomposites are marked with grey.

Wavenumber (cm^−1^)	Assignment [43,44,45]
517	Amine in-plane deformation
610	Ring deformation
720	Imine deformation
818	Amine deformation (C-N-C bending)
868	Ring deformation
1180	C-H bending in leucoemeraldine
1418	C-C stretching in quinoid-type ring
1595	C-C stretching in quinoid-type ring
1625	C-C stretching in benzene-type ring

**Table 2:** Characteristic IR bands of PANI. The bands appearing on the spectra of the nanocomposites are marked with grey.

Wavenumber (cm^−1^)	Assignment [46,47]
515	Aromatic ring deformation
828	C-H out-of-plane bending benzenoid-type ring
882	C-H out-of-plane bending
1070	NH_2_ rocking, ring deformation
1146	C-H in-plane-bending, ring deformation
1246	C-N stretching
1304	C-N stretching
1485	Benzenoid-type ring stretching
1568	Quinoid-type ring stretching
1610	N-H bending

## Abbreviations

The following abbreviations are used in this manuscript:

ATR-FT-IR: Attenuated Total Reflectance Fourier Transformed Infrared Spectroscopy
CP: Conducting Polymer
HR-TEM: High Resolution Transmission Electron Microscopy
NP: Nanoparticle
PANI: Polyaniline
PPy: Polypyrrole
SC: Semiconductor
SEM: Scanning Electron Microscopy
TEM: Transmission Electron Microscopy
TGA: Thermogravimetric Analysis
TO: Transverse Optical
UV-vis: Ultraviolet-visible
